# Hypnotic State Modulates Sensorimotor Beta Rhythms During Real Movement and Motor Imagery

**DOI:** 10.3389/fpsyg.2019.02341

**Published:** 2019-10-22

**Authors:** Sébastien Rimbert, Manuel Zaepffel, Pierre Riff, Perrine Adam, Laurent Bougrain

**Affiliations:** ^1^Université de Lorraine, CNRS, Inria, LORIA, Nancy, France; ^2^Independent Researcher, Dambach-la-ville, France; ^3^Hemodialysis Department, University Hospital of Strasbourg, Strasbourg, France

**Keywords:** hypnosis, event-related synchronization, event-related desynchronization, motor imagery, stroke rehabilitation, sensorimotor beta rhythms

## Abstract

Hypnosis techniques are currently used in the medical field and directly influences the patient's state of relaxation, perception of the body, and its visual imagination. There is evidence to suggest that a hypnotic state may help patients to better achieve tasks of motor imagination, which is central in the rehabilitation protocols after a stroke. However, the hypnosis techniques could also alter activity in the motor cortex. To the best of our knowledge, the impact of hypnosis on the EEG signal during a movement or an imagined movement is poorly investigated. In particular, how event-related desynchronization (ERD) and event-related synchronization (ERS) patterns would be modulated for different motor tasks may provide a better understanding of the potential benefits of hypnosis for stroke rehabilitation. To investigate this purpose, we recorded EEG signals from 23 healthy volunteers who performed real movements and motor imageries in a closed eye condition. Our results suggest that the state of hypnosis changes the sensorimotor beta rhythm during the ERD phase but maintains the ERS phase in the mu and beta frequency band, suggesting a different activation of the motor cortex in a hypnotized state.

## 1. Introduction

Hypnosis has been the subject of many debates or misunderstandings and its definition has constantly changed over time with currents of ideas or trends (Salem and Bonvin, 2012). To define the state of hypnosis better, since the 1950s, researchers have been studying this phenomenon under experimental laboratory conditions and investigated the cognitive, social, and psychological impact of this practice (Oakley and Halligan, 2013). Based on its findings, hypnosis can be defined as an altered state of attention, receptivity, and concentration during which the hypnotized person is captured by a suggestion made by the hypnotist (Erickson, 1958; Malrewicz et al., 1986). In practice, hypnosis sessions usually begin with an induction procedure that leads participants into a state of mental absorption and an augmented response to the suggestion (Elkins et al., 2015). Induction and suggestion procedures allow subjects to imagine experiences and gestures as real events (Konradt et al., 2005).

Based on these findings, hypnosis can modulate cognitive processes (i.e., perceptual, motor, emotional) by producing observable behavioral changes and subjective experiences (Landry et al., 2017). For example, hypnosis leads the hypnotized person to relaxation, a change in perceptions of the body and/or the environment, an increased imagination, and careful control (Spiegel and Spiegel, 1978; Rainville et al., 2002; Oakley and Halligan, 2013). This procedure is already applied to reduce pain, manage stress, strengthen the immune system, and to manage emotional problems (Bryant et al., 2005; Kupers et al., 2005; Wood and Bioy, 2008; Elahi et al., 2013). In this article, we will focus on Ericksonian therapeutic hypnosis, which is already used in many hospitals or clinical centers for its benefits (Erickson et al., 1976). Indirect suggestion is a characteristic of the Ericksonian hypnosis school since the metaphor is used to transmit the suggestion indirectly, through the patient's imagination.

Several studies have also confirmed the relevance of this approach for motor rehabilitation (Wright, 1960; Raginsky, 1963; Crasilneck and Hall, 1970). More precisely, hypnosis is particularly interesting for anxious patients, negativists or patients with a relatively low motivation for recovery (Appel, 2003). Moreover, hypnotic suggestion allows multiple states to be induce (e.g., being relaxed while focusing on feelings usually perceived during a real movement), which appears to be particularly relevant when performing motor imagery (MI) (Konradt et al., 2005; Muller et al., 2012; Simpkins and Simpkins, 2012), i. e., the ability to imagine performing a movement without executing it (Neuper et al., 2005; Guillot et al., 2009). Nowadays, the MI task is fundamental in rehabilitation protocols after a stroke (Zimmermann-Schlatter et al., 2008; García Carrasco and Aboitiz Cantalapiedra, 2013), because it activates the motor cortex, resulting in better synaptic plasticity, and therefore enhances rehabilitation during the subacute phase (Sharma et al., 2006; Cincotti et al., 2012; Ang and Guan, 2015).

Thus, hypnosis seems to be a good practice to improve motor rehabilitation for patients, particularly by helping the person to perform MIs. This issue however, has to be investigated, and understanding the underlying brain phenomenon remains a mystery. Indeed, several studies reported a correlation between patients' suggestibility and neuronal activation but very few of them are interested in how the motor cortex responds during the hypnosis process (London et al., 1968; Roelofs et al., 2002; Elahi et al., 2013). Other studies showed the effect of a suggested paralysis on motor areas, but the hypnotic induction used was very specific and not adaptable for stroke rehabilitation (Roelofs et al., 2002; Haggard et al., 2004; Cojan et al., 2009, 2013). Interestingly, Muller et al. showed differences between hypnotic and normal states during a MI and suggested that hypnosis enhanced the motor control circuit engaged in the motor task by modulating the gating function of the thalamus (Muller et al., 2013). Unfortunately, no studies have yet confirmed their findings and revealed the effect of hypnosis on the electroencephalographic signal (EEG) of the motor cortex during motor tasks. The hypnosis technique could also alter the activity of the motor cortex.

The beta rhythm is well-known to be involved in sensorimotor processes. Under normal conditions, a voluntary movement or a MI are characterized by three distinct and easily identifiable phases in the EEG signal. Initially, when compared to a resting state, the movement preparation phase shows a gradual decrease of power both in the alpha (7–13 Hz) and beta (15–30 Hz) bands (Pfurtscheller and Neuper, 1997). This is referred to as an event-related desynchronization (ERD) (Pfurtscheller and Aranibar, 1979; Pfurtscheller and Lopes da Silva, 1999). During movement or the motor imagery, a minimal power level is maintained in both bands (Pfurtscheller and Lopes da Silva, 1999; Cheyne, 2013). Finally, 300–500 ms after the end of the task, there is an increase of power referred to as an event-related-synchronization (ERS) in the beta band, also known as post-movement beta rebound, lasting ~1 s (Salenius et al., 1997; Cheyne, 2013; Kilavik et al., 2013). Concurrently, in the alpha band, the power returns to a baseline after several seconds. Typically, these patterns occur in the motor cortex, in the contralateral hemisphere (Pfurtscheller and Aranibar, 1977; Pfurtscheller and Neuper, 2001; Bai et al., 2005) but can appear bilaterally (Pfurtscheller et al., 1996; Fok et al., 2011; Formaggio et al., 2013). ERD and ERS patterns are also observed during passive movement (Müller et al., 2003), observed movement (Avanzini et al., 2012), kinesthetic illusion (Keinrath et al., 2006), or a median nerve stimulation (Salenius et al., 1997), raising fundamental questions about their sensorimotor role. To our knowledge, the impact of hypnosis on the EEG signal during movement or imagined movement has not been investigated. In particular, how ERD and ERS patterns are modulated for motor tasks may provide a better understanding of the potential benefits of hypnosis for stroke rehabilitation.

The goal of our study is to evaluate how hypnosis affects EEG signals in the motor cortex during two motor tasks (i. e., real movement and motor imagery). For this purpose, we recorded EEG signals from 23 healthy volunteers who performed motor tasks under normal and hypnotic conditions. We analyzed and compared the EEG modulations of the mu and the beta bands over the motor cortex for both conditions. The results suggest that hypnosis changes the sensorimotor beta rhythm during the ERD phase but maintains the ERS phase in the mu and beta frequency band, suggesting a different activation of the motor cortex in the hypnotized state.

## 2. Materials and Methods

### 2.1. Participants

Twenty-three right-handed healthy unpaid subjects (10 females; from 18 to 30 years-old with a mean and standard deviation age 26.1 years ± 2.8) were recruited for this experiment. This study is in accordance with the WMA declaration of Helsinki on ethical principles for medical research involving human subjects (World Medical, 2002). In addition, participants signed an informed consent form, which was approved by the ethical committee of Inria (the COERLE, approval number: 2016-011/01) as it satisfied the ethical rules and principles of the institute. The subjects had no medical history and no experience with hypnosis which could have influenced the task. All were normotensive (BP <140/90 mmHgl) and none were taking any cardiovascular medications or reported any history of neurological disorders.

### 2.2. Experimental Tasks

The aim of this study was to evaluate the impact of a hypnotic state on psychological and neurophysiological parameters during two motor tasks: (1) real movement (RM) and (2) motor imagery (MI). For the RM, the goal was to perform a simple sustained closing right hand-movement (Nakayashiki et al., 2014). For the MI, the goal was to imagine performing the same movement without executing it (Avanzini et al., 2012; Liang et al., 2016).

### 2.3. Experimental Environment

The experiment took place in a quiet confined room and was conducted by two researchers. One researcher assumed the technological process of the experiment. The second operator was a professional hypnotherapist in charge of instructing the subjects under both normal and hypnotic conditions. During the experiment, the subjects sat comfortably on a chair, with their right arm leaning on an armrest, their hand resting on an ergonomic pillow, without any muscular tension.

### 2.4. Experimental Design

**Figure 1:**
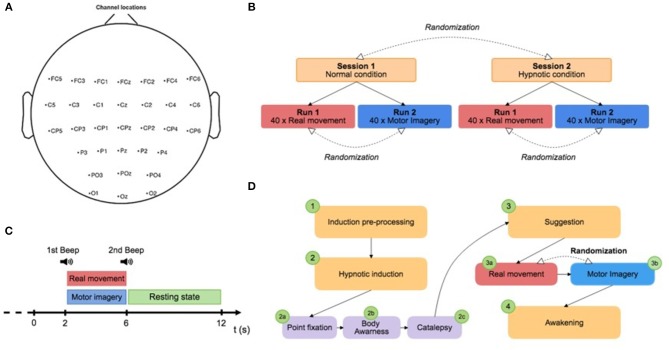
**(A)** Electrode position. **(B)** Paradigm scheme representing the two different sessions: normal condition and hypnotic condition. Each session is composed of two runs of 8 min and a randomization of the order was applied between runs and sessions. **(C)** Trial design: the subject had to perform a real movement task or a motor imagery task for 4 s, approximately once every 10 s according to triggers. A low frequency beep indicated when the subject had to execute the task (RM or MI). A high frequency beep indicated the end of the task. **(D)** Description of the hypnosis session. The hypnotic condition (Session 2) was subdivided into several stages: (1) the induction pre-process, (2) the hypnotic induction, (3) suggestion during the experiment, and (4) the awakening.

The protocol contained two sessions (normal condition and hypnotic condition) split in two different runs (real movement and motor imagery) that were completed by the subject on the same day. The sessions were performed in a counterbalanced order determined by computerized randomization. Sessions 1 and 2 correspond to the normal and hypnotic conditions, respectively (Figure 1B). For each session, one run of 8 min of real movements and one run of motor imageries were performed in a randomized order. During a run, the subject had to perform a real movement task or a motor imagery task for 4 s, approximately once every 10 s according to triggers. A low frequency beep indicated when the subject had to execute the task (RM or MI) (Figure 1C). A high frequency beep indicated the end of the task. At the beginning of each run, the subject remained relaxed for 10 s. Breaks of a few minutes were planned between runs and sessions to prevent fatigue of the subject. Before the experiment, both tasks (RM and MI) were previously described to the subject and the subject trained to master them. The subjects were instructed to avoid swallowing or other movements during the recording phases.

### 2.5. Hypnotic Procedure

The induction procedure was performed by a professional hypnotist. For each subject, a standard script written in advance and based on Ericksonian hypnosis was used. The hypnotic condition (Session 2) was subdivided into several stages: (1) the induction pre-process, (2) the hypnotic induction, (3) suggestion during the experiment, and (4) the awakening (Figure 1D). First, the induction treatment phase consisted of explaining hypnosis with simple words to make a connection with the subject. Second, the hypnotic induction was composed by three steps : (2a) the point fixation, (2b) the body awareness, and (2c) the catalepsy. The point fixation phase (2a) consisted of asking the subject to fix a point to locate his attention and improve his receptivity to the hypnotist's inductions. The awareness phase (2b) of the body helped to increase the subject's body awareness. Specifically, particular attention was paid to the breathing, the position of the body on the chair, the room temperature, and the intensity of the hypnotist's voice. The aim of the catalepsy step (2c) was to consolidate the hypnotic state with a “lever” technique in order to obtain a deep trance. Then, the experiment consisted of two runs (RM and MI). The subject began randomly with one of them to avoid bias. Between the two runs, the hypnotist used a new step of catalepsy to maintain the state of hypnosis. Finally, the awakening phase allowed the subject to return to the normal state.

### 2.6. Personality and Cognitive Profile Assessment Using Questionnaires

At the beginning of the experiment, participants completed a psychometric questionnaire to assess different aspects of their personality and cognitive profile. Then, all the subjects completed the French version of Movement Imagery Questionnaire - Revised Second Version (MIQ-RS) (Butler et al., 2012; Loison et al., 2013), which allowed for evaluation of the subject's ability to realize visual or kinesthetic motor imaginations. At the end of each session, participants completed the same questionnaire based on a Lickert scale to assess the possible impact of hypnosis on several criteria: estimated time, body perception, memory, detachment, stress, fatigue, and motivation.

### 2.7. Physiological Recordings

EEG signals were recorded through the OpenViBE platform with a commercial REFA amplifier developed by TMS International. The EEG cap was fitted with 32 electrodes re-referenced with respect to the common average references across all channels over the extended international 10-20 system positions. The selected electrodes were *FC*_5_, *FC*_3_, *FC*_1_,*FC*_*z*_, *FC*_2_, *FC*_4_, *FC*_6_, *C*_5_,*C*_3_, *C*_1_, *C*_*z*_, *C*_2_, *C*_4_, *C*_6_, *CP*_5_, *CP*_3_, *CP*_1_, *CP*_*z*_, *CP*_2_, *CP*_4_, *CP*_6_, *P*_3_, *P*_1_, *P*_*z*_, *P*_2_, *P*_4_, P*O*_3_, P*O*_*z*_, P*O*_4_, *O*_1_, *O*_*z*_, *O*_2_ (Figure 1A). These sites are localized around the primary motor cortex, the motor cortex, the somatosensory cortex and the occipital cortex, which allowed us to observe the physiological changes due to the RM and MI tasks. For this study, an external electromyogram (EMG) surface electrode was positioned on the right forearm of subjects for two reasons. First, we wanted to confirm that no movement was performed during the motor imagination task. In fact, based only on the experimenter's observation, it is very difficult to ensure that no micromovement is performed during the MI task. EMG activity during MI confirmed that there was no muscle activity during this mental task. Second, we needed to be able to test whether the real movements performed were similar to the normal condition despite the hypnotic state. Indeed, it has previously been shown that hypnotic suggestions may influence a movement strength, while increasing corticospinal excitability (Takarada and Nozaki, 2014). Respiratory rate and heart rate were measured with a Datascope Passport2 developed by Mindray. Impedance was kept below 5 kΩ for all electrodes to ensure that background noise in the acquired signal was low. No additional filtering was used during the recording.

### 2.8. Signal Pre-processing

All offline analyses were performed using the EEGLAB toolbox (Delorme and Makeig, 2004) and MATLAB 2016a (The MathWorks Inc. Natick, MA, USA). The data were processed in the General Data Format (GDF). Raw EEG data were resampled at 256 Hz, high-pass filtered at 0.5 Hz using a FIR filter and divided into 8 s epochs, corresponding to 1 s before and 7 s after the motor task for each run. Then, the EEG signal baseline was defined 2 s before each trial, therefore, a specific baseline was chosen for the two sessions. The results were also visualized by applying a Laplacian filter (Perrin et al., 1989) and confirmed those described below using the CAR method (Figure S1). Finally, based on the EMG activity, we removed trials that did not perform properly in the MI condition due to the presence of a micromovement or a real movement (Figure S2). For this purpose, we used the EMG electrode present throughout the experiment. We also eliminated trials when the EEG activity significantly exceeded ±50 μV, suggesting potential artifacts (Verleger, 1993; Moretti et al., 2003). The number of trials removed for each motor task and subject is described in Figure S3.

### 2.9. EMG Processing

The data from the EMG signal were resampled at 256 Hz, band-pass filtered between 5 and 450 Hz at order 4 (with a butterworth) and divided in 4 s epochs, corresponding to the motor task periods that appears between the two beeps. Then the root-mean-square (RMS) envelope was calculated for each subject for a 125 ms sliding window without overlapping (described in Farfán et al., 2010 (**Figure 3**). A grand average over all subjects (*n* = 23) was computed using values of the normal condition as a reference to normalize the signal of the hypnotic condition. The RMS represents the square root of the average power of the EMG signal for a given period of time. The RMS calculation is considered to have the largest information on the amplitude of the EMG signal because it provides a measure of the signal strength, while producing a waveform that is easily analyzed (Fukuda et al., 2010).

### 2.10. Time-Frequency Analysis

To analyze the differences between each session (normal condition vs. hypnotic condition), we performed an event-related spectral perturbation (ERSP) between 8 and 35 Hz. We computed the ERSPs using the gain model approach (Grandchamp and Delorme, 2011) which is equivalent to the “band power method” (Pfurtscheller and Lopes da Silva, 1999). In this model, the event-related spectral power at each time-frequency point is divided by the average spectral power in a 2 s pre-stimulus baseline period for each frequency band. ERSP visualizes event-related changes in the averaged power spectrum relative to a baseline interval taken 2 s before each trial (Brunner et al., 2013). Then, a log-transformed ERSP measure was used to highlight our results (1). The mean ERSP for frequency *f* and time point *t* is defined as

(1) $$\begin{array}{r} ERSP_{log}\left(f,t\right)=10\log_{10}\left(ERSP_{\%}\left(f,t\right)\right) \end{array}$$

We used a 256-point sliding fast Fourier transform (FFT) window with a padratio of 4 and we computed the mean ERSP 1 s before the task to 7 s after the task (**Figure 4**). The ERSPs were computed separately for all EEG channels and then observed in the alpha and beta frequency bands. Since ERD and ERS are difficult to observe we used the averaging technique to represent the modulation of power for both conditions (Pfurtscheller, 2003; Quiroga and Garcia, 2003).

### 2.11. Topographies

Brain topography allows us to display the possible changes over different electrodes on the scalp in order to localize which part of the brain was involved when the subject performed the requested task. We computed the topographic ERSPs in the beta (15–30 Hz) band for the normal condition and hypnotic condition (**Figure 5**).

### 2.12. Statistical Analysis

For the EMG processing, we chose to apply a paired *t*-test (two-sided) to show the significant difference between both conditions (*p*-value = 0.0011).

A surrogate permutation test (*p* < 0.05; 3,000 permutations) from the EEGLAB toolbox was used to validate differences in term of time-frequency ERSPs and localization of this ERSPs with a good alpha level (<5%) (**Figures 4**–**6**). In addition to this analysis, we applied a false discovery rate (FDR) correction test in order to clarify how the false discovery rate was controlled for multiple comparisons. This test performed the drawing of data samples without replacement (Manly, 2006).

**Figure 2:**
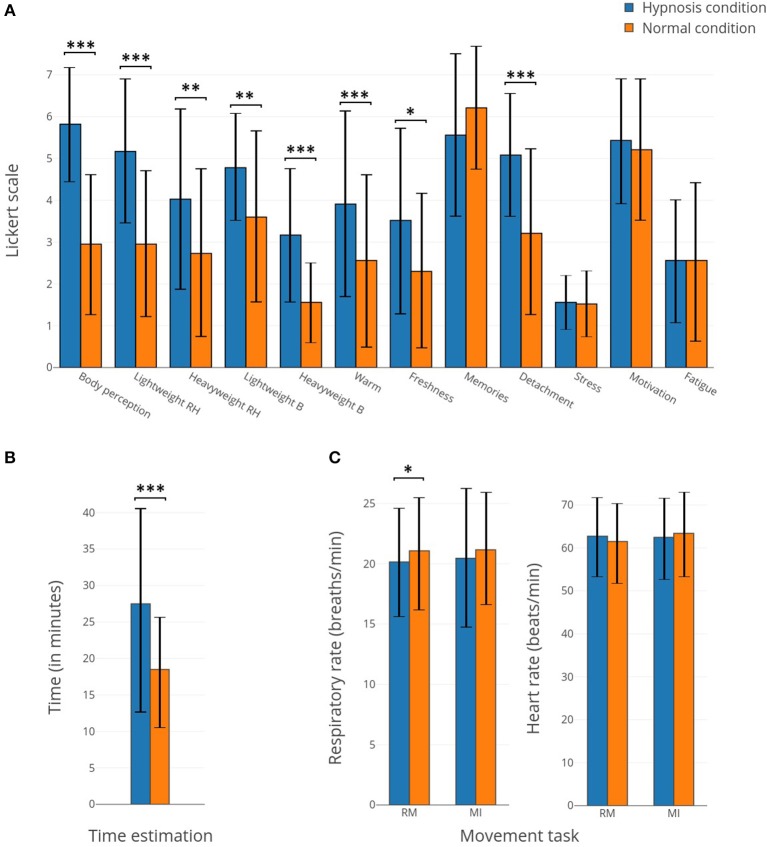
**(A)** Bar plots representing the responses to the questionnaires concerning the 12 items related to the subject's perceptions for hypnotic (in blue) and normal (in orange) conditions. **(B)** Bar plots representing the time estimation for hypnotic (in blue) and normal (in orange) conditions. **(C)** Bar plots representing the respiratory rate and heart rate for hypnotic (in blue) and normal (in orange) conditions. *Corresponds to a *p*-value <0.05, **corresponds to a *p*-value <0.01 and ***corresponds to a *p*-value <0.001.

We chose to apply a paired *t*-test (two-sided) to show the significant difference about questionnaire variations (Figure 2; *p*-value < 0.01).

Then, considering the use of Likert scales in the questionnaires, we computed Spearman's correlation to establish a correlation between questionnaires and ERD/ERS variations between the normal and hypnotic state (**Figure 6A**; *p*-value < 0.05).

## 3. Results

The findings of this research can be defined as both behavioral and electrophysiological. First, the findings are of a behavioral nature, with the analysis of both the pre- and post-experimentation questionnaires and the EMG signal. Second, the findings are also of a electrophysiological nature, with the analysis of ERD and ERS modulations.

### 3.1. Behavioral Results

#### 3.1.1. Questionnaires

After each (normal or hypnotic) condition, subjects filled in a post-experimentation questionnaire. The studied items were modification of body perception, lightness/heaviness of the right arm, lightness/heaviness of the body, heat/fresh sensation, event memorization, feelings of detachment, stress, motivation, and tiredness. In addition to these variables, the participants had to estimate for how long they had performed the condition while the duration for all runs was the same. Most of the perception items show significant differences between the normal and hypnotic conditions (Figure 2A). The body perception, light weight of the right hand, heavy weight of the body, warmness and detachment sensation show the biggest differences (*p*-value < 0.001). In addition, subjects estimated a longer duration for the hypnotic state (*p*-value < 0.01). The respiratory rate and heart rate show small differences, except for the respiratory rate during the RM which displays a small but significant difference.

#### 3.1.2. Movement Analysis

The root-mean-square (RMS) envelope of the EMG signal for real movements shows an average difference of 14.9% between normal and hypnosis conditions. A student *t*-test was performed on the 40 trials for the 23 subjects, and this difference is significant (*p*-value = 0.0011). For the MI task, no significant EMG activity was detected.

### 3.2. Electrophysiological Results

#### 3.2.1. ERD and ERS Modulations During Normal Condition

For the RM task (**Figure 4B**), an ERD appears 250 ms after the cue both in the mu band and the beta band, last for 3,250 ms, and stops 500 ms before the second cue. Then, an ERS appears in the beta band at 4,000 ms and last until 6,500 ms.

For the MI task (**Figure 4D**), the ERD is present from 250 to 3,000 ms and is mostly present in the beta band. There is no ERS in the beta band after the second cue, but a small one is present in the mu band (from 4,000 to 6,250 ms).

Concerning the topographic maps for these two tasks (**Figure 5**), the RM task shows a bilateral distribution of the ERD, centered around electrodes C3 and C4. The ERS is present on the C3 and FC2 electrodes. For the MI task, the ERD is mostly contralateral and centered on the C3 electrodes, the ERS is also mostly contralateral.

#### 3.2.2. ERD and ERS Modulations During Hypnosis Condition

During the hypnotic condition, for the RM task (**Figure 4A**) we observed a smaller ERD than in the normal condition. It started at the same time, 250 ms after the cue, but quickly disappeared around 750 ms, and was only present on the beta band during this time window. The ERD reappeared at 2,000 ms in the band and disappeared at 3,500 ms. The ERS was mostly present in the beta band from 4,000 to 6,500 ms. There was a small ERS in the mu band from 5,000 to 6,000 ms.

For the MI task (**Figure 4C**), a small ERD appeared on both frequency bands from 250 to 1,000 ms. It was then replaced by an ERS on the beta band from 1,000 to 2,500 ms. Another ERD then appeared at 2,500 ms in the beta band (mostly between 14 and 16 Hz) and quickly disappeared 250 ms later. An ERS then took over on the beta band at 3,000 ms and lasted up to 5,500 ms.

Concerning the topographic map (**Figure 5**), for the RM task the ERD is bilateral and centered around electrodes C3 and C4. The ERS is mostly left-sided around C3. During the MI task, the ERD are also centered around electrodes C3 and C4, and the ERS was mostly centered around telectrode C3 from 4,000 to 5,000 ms.

#### 3.2.3. Statistical Differences Between the Hypnotic Condition and the Normal Condition

For the RM task the absence of ERD from 0 to 2,000 ms in the beta band is the main significant difference (**Figure 4**). For the MI task, the statistical differences are mostly present on the beta band during most of the task duration, the absence of ERD during the task on the hypnotic condition, and the presence of an ERS is reflected in the statistical analysis. We can also observed a significant difference at 4,000 ms in the alpha band, explained by the presence of an ERS in the hypnotic condition.

#### 3.2.4. Effect of the Randomized Sequence Order on the ERD

We also checked that there was no bias resulting from the sequence in which the conditions (hypnotic vs. normal) were achieved for each subject. Indeed, participants who started with the hypnotic condition may have a long-term effect influencing the next normal condition. **Figure 6** indicates that there is no significant difference (at *p*-value < 0.05) in the ERD phase depending on the condition or task the subject started with, although subjects who started with the normal condition and the MI task seem to have minimal ERD.

### 3.3. Correlation Between Questionnaires and EEG

No significant correlation was found between the post-experiment questionnaire results and ERD and ERS patterns. We tested the correlation by considering all the items contained in the questionnaire (**Figure 6**). Only heat, freshness and detachment could be correlated with ERD and ERS variations (at *p*-value < 0.05). Most of the significant correlations have a value between 0.5 and 0.75 which means that every shift in perception was positively correlated with the other, as expected if the hypnosis induction was correctly performed on the subject.

## 4. Discussion

In this study, we used the EEG technique to investigate whether a hypnotic suggestion impacted the ERD and ERS modulations that occur during the execution of a voluntary actual or imagined movement. The hypnotic suggestion used was made specifically to enhance the sensory feelings perceived during the two motor tasks. Our results show that the hypnosis technique significantly altered both the subject's subjective perceptions during the experiment and the ERD and ERS modulations resulting from motor tasks in the EEG signal. In this section, we will first discuss how behavior was modified with hypnosis. Second, we will analyze ERD and ERS modulations during the normal and hypnotic conditions. Finally, we will discuss the mechanism of the motor cortex activity under hypnosis and the potential impact of these findings for motor rehabilitation and the brain-computer interface (BCI) domain.

### 4.1. Hypnosis Effect on Behavior

#### 4.1.1. Subjective Perceptions During Hypnosis

Altogether, our results reveal that hypnosis significantly alters many subjective perceptions, providing relative evidence of the effectiveness of hypnotic suggestion (Figure 2). Indeed, compared to a normal state, the subjects reported a modified perception of their body, alterations of body temperature and forearm weight corresponding to an increase or a decrease depending on the subject. With the hypnosis technique, such feelings that appear contradictory are possible (Simpkins and Simpkins, 2012). Similar reports were also found in other studies (Kihlstrom, 2008; Landry et al., 2017). Some perceptions experienced in a hypnotic state may be considered contradictory (e.g., freshness and warmth of the body, heaviness and lightness of the body, and more specifically of the right arm) but during the post-experiment testimonies, participants confirmed that multiple sensations were felt and varied with the moment of the experience. In addition, with hypnosis, subjects became more disengaged from the experiment and presented an upward time distortion (Figure 2). Time distortion in hypnosis is quite common but some studies suggest that subjects underestimate time during hypnosis (Naish, 2003; Haggard et al., 2004) while others overestimate it (Mozenter and Kurtz, 1992). No change can be observed in terms of stress, motivation, fatigue, or the memorization of events. There is also no evidence of any effect on the respiratory rate and/or heart rate, although it has previously been shown that hypnosis may have an effect on both parameters (Yuksel et al., 2013). However, in this study, the suggestion was centered on the increased sensations during motor tasks and was not essentially focused on relaxation, which could explain why these two parameters remained unchanged during the hypnosis state.

#### 4.1.2. EMG Activity During Hypnosis

**Figure 3:**
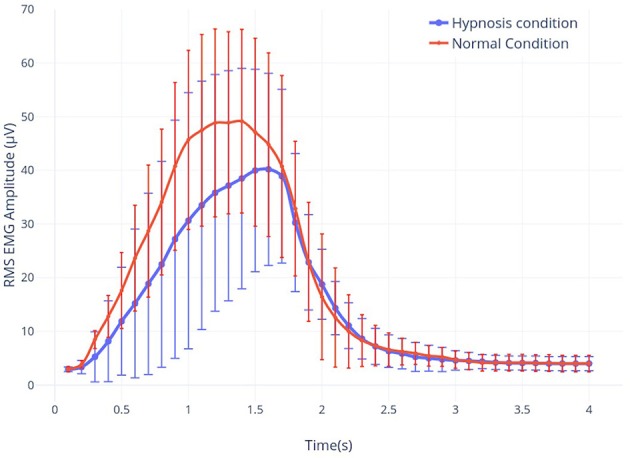
The root-mean-square (RMS) envelope of the EMG signal for real movement during both hypnosis (in blue) and the normal condition (in red).

Our results highlight that the movement performed under hypnosis was slightly different from the one performed in real movement (Figure 3). Indeed, muscle contraction was reduced (−14.9%), reflecting the impact of the hypnotic condition on behavior.

### 4.2. ERD and ERS Modulations Under Hypnosis

In this article, we aimed to study EEG motor activity of two motor tasks (i.e., real and imagined movements) in a normal condition and after a suggestion of hypnosis. For this purpose, we have chosen to analyze the modulations of ERD and ERS that are usually present at the different stages of a real or imagined movement.

#### 4.2.1. Motor Responses Without Hypnosis

To understand how hypnosis impacts ERD and ERS modulations, results obtained under the normal condition need to be compared with existing studies. Our results are consistent with the literature, highlighting the reliability of the motor brain responses obtained for both motor tasks.

##### 4.2.1.1. Real movement task

**Figure 4:**
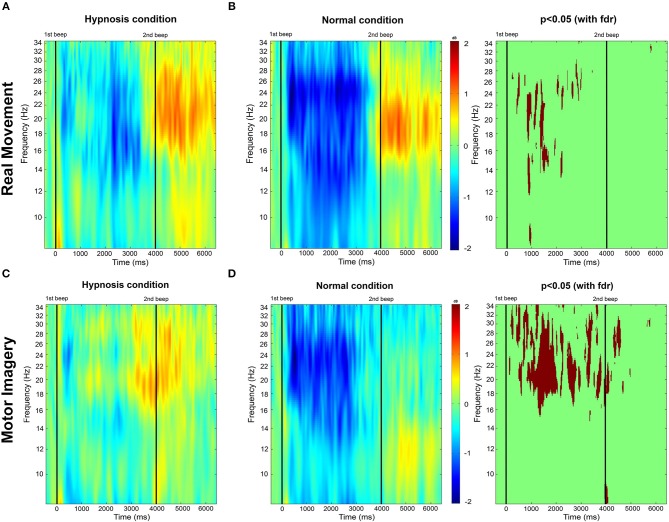
**(A,B)** Time-frequency grand average analysis corresponding to an event-related spectral perturbation (ERSP) for Session 1 (Normal condition) and Session 2 (Hypnotic condition) for a real movement for electrode *C*_3_. **(C,D)** Time-frequency grand average analysis (ERSP) for Session 1 (Normal condition) and Session 2 (Hypnotic condition) for a motor imagery for electrode *C*_3_. Red corresponds to a strong ERS and blue to a strong ERD. Significant differences (*p* < 0.05) with a False Discovery Rate (FDR) correction are shown in the right part of the figure.

Indeed, during a real movement, in a normal condition, Kilavik et al. showed that the power of the beta rhythm was very reduced during the dynamic phases of movement (Kilavik et al., 2013). In agreement with previous findings, we could identify two consecutive ERD peaks which correspond to muscle contraction and relaxation, respectively. Our results show clearly that this decrease is characterized by an ERD during all motor executions (Figures 4A,B). A first ERD peak starts 200 ms after the first sound beep (see Figures 4A,B). In this article, the cue used was a sound beep emitted every 8–12 s (with a random delay of 2 s centered around 10 s), which implies that the movement is not completely voluntary and not too prepared. As a consequence, no preparatory phase prior to motor performance can be identified, which has already been shown in previous articles (Alegre et al., 2006; Rimbert et al., 2018, 2019b). A second peak was observed at 2,500 ms, suggesting that the subjects anticipated the end of the real motor execution. This hypothesis is supported by the analysis of the EMG activity (Figure 3) and is probably explained by the difficulties of maintaining motor performance for 4 s throughout the experiment. Typically, the end of the movement is followed by a predominant ERS between 16 and 22 Hz, better known as beta, rebound (Pfurtscheller et al., 1997; Kilavik et al., 2013).

**Figure 5:**
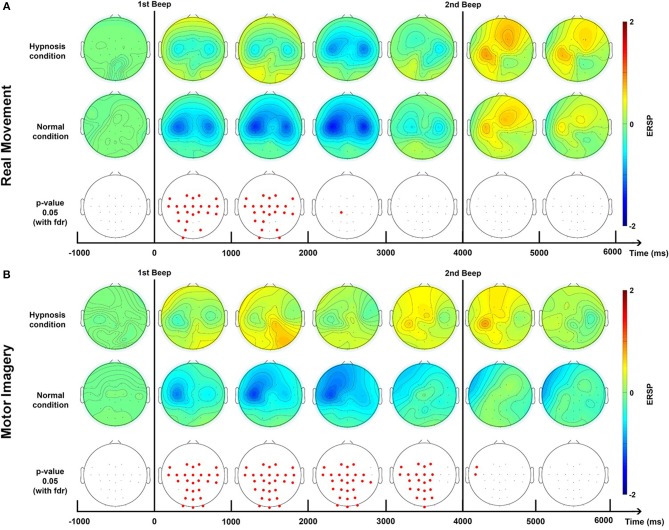
Topographic maps of ERD/ERS% (grand average, *n* = 23) in the Beta band (15–30 Hz) for a real movement **(A)** and a motor imagery **(B)** during two conditions: normal and hypnotic. Red corresponds to a strong ERS and blue to a strong ERD. The first vertical black line indicates when the motor task started and the second one to when it stopped. This figure is an extrapolation of 32 electrodes. Red electrodes indicate a significant difference (*p* < 0.05) with an FDR correction.

Topographic analysis (Figure 5) shows that the ERD is bilateral during a real movement while the post-movement ERS phase is contralateral, as previously established (Pfurtscheller and Aranibar, 1979; Bai et al., 2005).

In our task, the muscular contraction has to be maintained until the second beep. According to previous studies (Kilavik et al., 2013), a sustained movement goes with an increase in beta power. Even if beta power seems to increase between the two ERD peaks which correspond to the dynamic phase of the movement, the ERD is rather low. It may be due to the short delay between the two ERD peaks and the existence of preparatory power decrease related to the preparation of movement relaxation in a way that no ERS related to the sustained contraction can arise.

##### 4.2.1.2. Motor imagery task

In a normal condition, the ERD of a MI is relatively similar to the one obtained with a RM but with a smaller amplitude. This is a very well-known observation. The rebound is also attenuated, or even almost non-existent (Figures 4C,D). Several studies have already shown that the intention to move generates modulations of ERD and ERS of smaller amplitudes compared to real movement, due to the activation of similar neural networks at a lower scale (Filgueiras et al., 2017). Again, it would seem that the MI was not maintained until the second beep, which may be explained by the difficulty of maintaining a MI for 4 s. Moreover, EMG also showed a premature end of muscular activity (Figure 3) Finally, in the literature it is common for the MI to generate a contralateral ERD during the task followed by an ERS (Pfurtscheller et al., 2005; Hashimoto and Ushiba, 2013; Clerc et al., 2016), which is apparent in our results (Figure 5). Altogether, for the non-hypnotic condition, our results are highly consistent with the literature for both RM and MI.

#### 4.2.2. Motor Responses With Hypnosis

##### 4.2.2.1. Real movement task

In the introduction we made the hypothesis that a hypnotic suggestion oriented toward the sensation and full consciousness of movement would enable a more powerful motor brain response, and therefore a more important ERD and ERS. In opposition to our hypothesis, the results show that hypnosis during real movement can significantly reduce ERD during motor performance (Figures 4A,B), while the rebound, both in terms of amplitude and delay, is unchanged after a hypnotic suggestion.

##### 4.2.2.2. Motor imagery task

For the MI task, hypnosis produces more changes. Indeed, the ERD is even more attenuated than in real movement. The rebound is also higher in terms of amplitude while latency is lower (Figures 4C,D). Hypnosis would therefore have a common effect for the two motor tasks: an attenuation of the ERD phase with a differential effect for MI, with a very pronounced rebound in the contralateral direction (Figure 5B). These results cannot be explained by reduced EMG activity under hypnosis. First, because several studies have shown that the strength of the movement has no impact on the ERD phase (Kilavik et al., 2013), and second, because the same modulation, i.e., attenuation of ERD, is also present with a motor imagery.

#### 4.2.3. Are ERD and ERS Changes Related to Differences in Subjective Perceptions?

**Figure 6:**
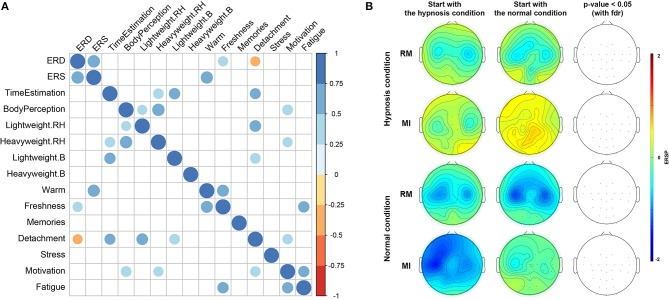
**(A)** Correlogram representing Spearman's correlations among the 12 items contained in the questionnaires, and ERD and ERS variation differences between normal and hypnotic conditions. Correlations with a *p*-value > 0.05 are considered as not significant and are shown in blank. Positive correlations are displayed in blue and negative correlations are in orange. The intensity of the color and the size of the circles are proportional to the correlation coefficients. **(B)** Topographic maps representing differences in term of ERSPs for the sequence in which the conditions (hypnotic and normal) were achieved for each subject. Red corresponds to a strong ERS and blue to a strong ERD. Significant difference (*p* < 0.05) with a False Discovery Rate (FDR) correction are shown in the right part of the figure.

Our results show that hypnosis has an effect on a subject's perception (e.g., time distortion, body feelings, detachment) but also on the EEG signal with a significant attenuation of the ERD phase. Then the following question was raised: can the differences in subjective perception experienced by the subjects during hypnosis explain the differences in modulations recorded during the ERD phase? In order to answer this question, the correlogram results indicate very low correlation between variations in ERD and ERS between normal and hypnotic conditions and the item values of the post-experiment questionnaire (Figure 6). Only heat, freshness, and detachment could be correlated with ERD and ERS variations.

However, the ERD variation is directly correlated (*p*-value < 0.05) to the ERS change, which, to our knowledge, has not been shown in previous studies.

### 4.3. Motor Cortex Mechanism Under Hypnosis

#### 4.3.1. Does Hypnosis Deactivate the Motor Cortex?

The absence of ERD during hypnosis for both motor tasks raises questions about the particular motor cortex mechanism in this altered state.

This reduction seems to be more important between the two peaks of the ERD, i.e., for the phase of sustained muscle contraction, but in fact with no significant difference (Figure 4A). Although it would appear that hypnosis leads to a global increase of power in the beta frequency band after the movement, our results for RM demonstrate that the rebound phase is not affected (Figures 4A,B).

Many authors have interpreted the ERD phase during a motor task as related to motor cortex activation (Pfurtscheller, 2001; Neuper et al., 2006), though it reflects the conjunction of multiple factors associated with sensory and cognitive aspects of motor control rather than pure motor processes (Kilavik et al., 2013). Thus, the significant attenuation of ERD for the two motor tasks under hypnosis would result from a weaker activation of the motor cortex. However, Muller et al. highlighted with fMRI that hypnosis could enhance the motor control circuit engaged in motor imagery by modulating the gating function of the thalamus (Muller et al., 2013). Furthermore, hypnosis may create a specific state that may enhance the efficiency of the motor system and also increase corticospinal excitability during movement (Takarada and Nozaki, 2014). Nevertheless, caution is required when studying hypnosis due to the variability between experiments, in particular in terms of the hypnotic induction used, the suggestion made, or the cognitive tasks performed by the subjects.

Increased automatism during hypnosis could explain an attenuation of the ERD phase despite the execution of the task (Déry et al., 2014). Indeed, in hypnotic states, subjects demonstrate extraordinary obedience by producing a movement in response to a suggestion that is described as an ideomotor response (Haggard et al., 2004). Finally, under hypnosis, the motor cortex may be less involved to the benefit of other more internal structures, such as the thalamus, which is considered the location of a high subjective experiences (Raz et al., 2003; Muller et al., 2013).

#### 4.3.2. Use of Hypnosis for Stroke Rehabilitation

Nowadays, the MI task is fundamental in rehabilitation protocols after a stroke (Zimmermann-Schlatter et al., 2008; García Carrasco and Aboitiz Cantalapiedra, 2013). The MI task activates the motor cortex, stimulating the injured area and all the evidence suggests that there is an impact on synaptic plasticity, resulting in better rehabilitation during the subacute phase (Liu et al., 2004; Cincotti et al., 2012; Pichiorri et al., 2015). Interestingly, hypnosis has an effect on relaxation, concentration, or increased perception but is also particularly relevant for imaginative procedures (Muller et al., 2012). Moreover, many studies have also confirmed the beneficial role it plays in motor rehabilitation (Wright, 1960; Crasilneck and Hall, 1970), so we expected an increase of the ERD during the motor tasks (Figures 4A,D). Consequently, our results prompt us to be cautious about using the hypnosis technique since the suggestion used could be unproductive. In this study, the suggestion used by the hypnotherapist was to feel as many feelings as possible during the motor tasks, and a majority of subjects reported an easier way of imagining movement compared to the normal condition. Despite this positive feedback, our results showed that hypnosis involves a vanishing of the ERD during the RM and MI tasks, which implies a lower activation of the motor cortex or an activation according to other modalities. Further research investigating the EEG connectivity index would be interesting to measure possible changes in the beta sensorimotor rhythm during hypnosis correlated with modulations related to the Default Mode Network (DMN). Indeed, several neurophysiological studies indicate that the state of hypnosis is associated with changes in connectivity (Fingelkurts et al., 2007; Jiang et al., 2016), especially in the DMN network (Lipari et al., 2011; Landry et al., 2014). Although, McGeown et al. have shown that the induction of hypnosis can reduce pre-rest DMN activity without increasing activity in other cortical regions (Mcgeown et al., 2009). To the best of our knowledge, only a few studies on the connectivity during motor tasks under hypnosis have been conducted. A new study combining EEG and fMRI would explore this issue and provide better guidance for the use of hypnosis practice for stroke rehabilitation.

#### 4.3.3. Hypnosis in the BCI Domain

Brain-computer interfaces allow end users to interact with a system using modulations of brain activities (Jonathan Wolpaw, 2012). Most of these BCIs are based on the modulation of sensorimotor rhythms which are partially observable in EEG signals during a MI task. In order to improve BCI performance and allow a better control of MI, it has been suggested that some altered states of consciousness, such as mind-body awareness training (MBAT) (Tan et al., 2014) or hypnosis (Alimardani and Hiraki, 2017) can be used, especially during the training phase. Interestingly, the hypnotic suggestion allows multiple states to be induced (e.g., being relaxed while focusing on the feelings usually perceived during a real movement), which appears to be particularly relevant to the MI task (Konradt et al., 2005; Simpkins and Simpkins, 2012). Recently, we investigated this question in a study which shows that the attenuation of ERD induced by hypnosis had a negative impact on BCI performance. This prompts us to not recommend using hypnosis for BCI applications (Rimbert et al., 2019a).

## 5. Conclusion

In this study, we investigated the impact of hypnosis on the sensorimotor beta rhythm for two motor tasks (i.e., real movement and motor imagery). We conducted an experiment on 23 subjects, using both motor tasks under two different conditions: hypnosis and normal state. The hypnotic induction was Ericksonian, essentially based on relaxation, and the suggestion was aimed at improving feeling and focus during movement and motor imagery.

This work showed that the state of hypnosis changes the sensorimotor beta rhythm during the ERD phase but maintains the ERS phase in the mu and beta frequency band suggesting a different activation of the motor cortex in the hypnotized state. In addition, the subjects perception and behavior was changed during the hypnotic condition. These findings raise questions about the use of this technique for rehabilitation after a stroke or in the BCI domain.

## Data Availability Statement

The datasets generated for this study are available on request to the corresponding author.

## Ethics Statement

The studies involving human participants were reviewed and approved by Ethical Committee of Inria (the COERLE). The patients/participants provided their written informed consent to participate in this study.

## Author Contributions

SR, MZ, and PA conceived and designed the experiments, performed the experiments. SR, MZ, PR, PA, and LB analyzed the data, contributed reagents, materials, and analysis tools, prepared figures and tables, authored or reviewed drafts of the paper, approved the final draft.

### Conflict of Interest

The authors declare that the research was conducted in the absence of any commercial or financial relationships that could be construed as a potential conflict of interest.

## Supplementary Material

Figure S1 Time-frequency grand average with Laplacian filter analysis corresponding to an event-related spectral perturbation (ERSP) for Session 1 (Normal condition) and Session 2 (Hypnotic condition) for a real movement and a motor imagery for electrode C3. Red corresponds to a strong ERS and blue to a strong ERD. Significant differences (*p* < 0.05) with a False Discovery Rate (FDR) correction are shown in the right part of the figure.

Figure S2 The root-mean-square (RMS) envelope of the EMG signal for real movement and motor imagery (dashed line) during both hypnosis (in blue) and the normal condition (in red).

Figure S3 Number of trials removed for each subject for real movement and motor imagery during hypnosis and normal condition.

## References

[B1] Alegre M. Imirizaldu L. Valencia M. Iriarte J. Arcocha J. Artieda J. (2006). Alpha and beta changes in cortical oscillatory activity in a go/no go randomly-delayed-response choice reaction time paradigm. Clin. Neurophysiol. 117, 16–25. 10.1016/j.clinph.2005.08.030 16316781

[B2] Alimardani M. Hiraki K. (2017). Development of a real-time brain-computer interface for interactive robot therapy: an exploration of eeg and emg features during hypnosis. Int. J. Comput. Electric. Autom. Control Inform. Eng. 11, 187–195. 10.5281/zenodo.1340062

[B3] Ang K. Guan C. (2015). Brain-computer interface for neurorehabilitation of upper limb after stroke. Proc. IEEE 103, 944–953. 10.1109/JPROC.2015.2415800

[B4] Appel P. (2003). Clinical hypnosis in rehabilitation. Semin. Integr. Med. 1, 90–105. 10.1016/S1543-1150(03)00010-3

[B5] Avanzini P. Fabbri-Destro M. Dalla Volta R. Daprati E. Rizzolatti G. Cantalupo G. (2012). The dynamics of sensorimotor cortical oscillations during the observation of hand movements: an EEG study. PLoS ONE 7:e37534. 10.1371/journal.pone.0037534 22624046 PMC3356327

[B6] Bai O. Mari Z. Vorbach S. Hallett M. (2005). Asymmetric spatiotemporal patterns of event-related desynchronization preceding voluntary sequential finger movements: a high-resolution EEG study. Clin. Neurophysiol. 116, 1213–1221. 10.1016/j.clinph.2005.01.006 15826864

[B7] Brunner C. Delorme A. Makeig S. (2013). EEGLAB–an open source matlab toolbox for electrophysiological research. Biomed. Tech. 58, 9–21. 10.1515/bmt-2013-4182 24042816

[B8] Bryant R. Moulds M. Guthrie R. Nixon R. (2005). The additive benefit of hypnosis and cognitive-bahvioral therapy in treating acute stress dirsorder. J. Consult. Clin. Psychol. 73, 334–340. 10.1037/0022-006X.73.2.334 15796641

[B9] Butler A. Cazeaux J. Fidler A. Jansen J. Lefkove N. Gregg M.. (2012). The movement imagery questionnaire-revised, second edition (MIQ-RS) is a reliable and valid tool for evaluating motor imagery in stroke populations. Evid. Based Complement. Alternat. Med. 2012:49728. 10.1155/2012/497289 22474504 PMC3304547

[B10] Cheyne D. (2013). MEG studies of sensorimotor rhythms: a review. Exp. Neurol. 245, 27–39. 10.1016/j.expneurol.2012.08.030 22981841

[B11] Cincotti F. Pichiorri F. Arico P. Aloise F. Leotta F. de Vico Fallani F.. (2012). EEG-based brain-computer interface to support post-stroke motor rehabilitation of the upper limb. Conf. Proc. IEEE Eng. Med. Biol. Soc. 2012, 4112–4115. 10.1109/EMBC.2012.6346871 23366832

[B12] Clerc M. Bougrain L. Lotte F. (2016). Brain-Computer Interfaces. Vol. 1, Foundations and Methods, Chapter EEG Feature Extraction. London: ISTE Ltd Hoboken, 130–131.

[B13] Cojan Y. Archimi A. Cheseaux Waber L. Vuilleumier P. (2013). Time-course of motor inhibition during hypnotic paralysis: EEG topographical and source analysis. Cortex 49, 423–436. 10.1016/j.cortex.2012.09.013 23211547

[B14] Cojan Y. Waber L. Schwartz S. Rossier L. Forster A. Vuilleumier P. (2009). The brain under self-control: modulation of inhibitory and monitoring cortical networks during hypnotic paralysis. Neuron 62, 862–875. 10.1016/j.neuron.2009.05.021 19555654

[B15] Crasilneck H. Hall J. (1970). The use of hypnosis in the rehabilitation of complicated vascular and post-traumatic neurological patients. Int. J. Clin. Exp. Hypn. 18, 145–159. 5484100 10.1080/00207147008415914

[B16] Delorme A. Makeig S. (2004). EEGLAB: an open source toolbox for analysis of single-trial EEG dynamics including independent component analysis. J. Neurosci. Methods 134, 9–21. 10.1016/j.jneumeth.2003.10.009 15102499

[B17] Déry C. Campbell N. K. Lifshitz M. Raz A. (2014). Suggestion overrides automatic audiovisual integration. Conscious. Cogn. 24, 33–37. 10.1016/j.concog.2013.12.010 24398260

[B18] Elahi Z. Boostani R. Nasrabadi A. (2013). Estimation of hypnosis susceptibility based on electroencephalogram signal features. Sci. Iran. 20, 730–737. 10.1016/j.scient.2012.07.015

[B19] Elkins G. Barabasz A. Council J. Spiegel D. (2015). Advancing research and practice: the revised apa division 30 definition of hypnosis. Int. J. Clin. Exp. Hypn. 63, 1–9. 10.1080/00207144.2014.961870 25365125

[B20] Erickson M. (1958). Hypnosis in painful terminal illness. Am. J. Clin. Hypn. 56, 67–71. 13664639

[B21] Erickson M. Rossi E. Rossi S. (1976). Hypnotic Realities: The Induction of Clinical Hypnosis and Forms of Indirect Suggestion. New York, NY: Irvington Publishers.

[B22] Farfán F. Politti J. Felice C. (2010). Evaluation of EMG processing techniques using information theory. Biomed. Eng. Online 9:72. 10.1186/1475-925X-9-72 21073705 PMC2989313

[B23] Filgueiras A. Quintas Conde E. Hall C. (2017). The neural basis of kinesthetic and visual imagery in sports: an ALE meta-analysis. Brain Imaging Behav. 12, 1513–1523. 10.1007/s11682-017-9813-9 29260381

[B24] Fingelkurts A. A. Fingelkurts A. A. Kallio S. Revonsuo A. (2007). Cortex functional connectivity as a neurophysiological correlate of hypnosis: an EEG case study. Neuropsychologia 45, 1452–1462. 10.1016/j.neuropsychologia.2006.11.018 17208259

[B25] Fok S. Schwartz R. Wronkiewicz M. Holmes C. Zhang J. Somers T.. (2011). An EEG-based brain computer interface for rehabilitation and restoration of hand control following stroke using ipsilateral cortical physiology. Conf. Proc. IEEE Eng. Med. Biol. Soc. 2011, 6277–6280. 10.1109/IEMBS.2011.6091549 22255773

[B26] Formaggio E. Storti S. Galazzo I. Gandolfi M. Geroin C. Smania N.. (2013). Modulation of event-related desynchronization in robot-assisted hand performance: brain oscillatory changes in active, passive and imagined movements. J. Neuroeng. Rehabil. 10:24. 10.1186/1743-0003-10-24 23442349 PMC3598512

[B27] Fukuda T. Y. Echeimberg J. O. Pompeu J. E. Lucareli P. R. G. Garbelotti S. Gimenes R. O. (2010). Root mean square value of the electromyographic signal in the isometric torque of the quadriceps, hamstrings and brachial biceps muscles in female subjects. J. Appl. Res. 10, 32–39.

[B28] García Carrasco D. Aboitiz Cantalapiedra J. (2013). Effectiveness of motor imagery or mental practice in functional recovery after stroke: a systematic review. Neurologia 31, 43–52. 10.1016/j.nrleng.2013.02.008 23601759

[B29] Grandchamp R. Delorme A. (2011). Single-trial normalization for event-related spectral decomposition reduces sensitivity to noisy trials. Front. Psychol. 2:236. 10.3389/fpsyg.2011.00236 21994498 PMC3183439

[B30] Guillot A. Collet C. Nguyen V. A. Malouin F. Richards C. Doyon J. (2009). Brain activity during visual versus kinesthetic imagery: an FMRI study. Hum. Brain Mapp. 30, 2157–2172. 10.1002/hbm.20658 18819106 PMC6870928

[B31] Haggard P. Cartledge P. Dafydd M. Oakley D. (2004). Anomalous control: when 'free-will' is not conscious. Conscious. Cogn. 13, 646–654. 10.1016/j.concog.2004.06.001 15336254

[B32] Hashimoto Y. Ushiba J. (2013). EEG-based classification of imaginary left and right foot movements using beta rebound. Clin. Neurophysiol. 124, 2153–2160. 10.1016/j.clinph.2013.05.006 23757379

[B33] Jiang H. White M. Greicius M. Waelde L. Spiegel D. (2016). Brain activity and functional connectivity associated with hypnosis. Cereb. Cortex 27, 4083–4093. 10.1093/cercor/bhw220 27469596 PMC6248753

[B34] Jonathan Wolpaw E. W. W. (ed.). (2012). Brain-Computer Interfaces: Principles and Practice. Oxford; New York, NY: Oxford University Press.

[B35] Keinrath C. Wriessnegger S. Muller-Putz G. R. Pfurtscheller G. (2006). Post-movement beta synchronization after kinesthetic illusion, active and passive movements. Int. J. Psychophysiol. 62, 321–327. 10.1016/j.ijpsycho.2006.06.001 16904786

[B36] Kihlstrom J. F. (2008). The domain of hypnosis, revisited, in Oxford Handbook of Hypnosis: Theory, research, and practice, eds Nash M. R. Barnier A. J. (New York, NY: Oxford University Press), 21–52.

[B37] Kilavik B. E. Zaepffel M. Brovelli A. MacKay W. A. Riehle A. (2013). The ups and downs of beta oscillations in sensorimotor cortex. Exp. Neurol. 245, 15–26. 10.1016/j.expneurol.2012.09.014 23022918

[B38] Konradt B. Deeb S. Scholz O. B. (2005). Motor imagery in hypnosis: accuracy and duration of motor imagery in waking and hypnotic states. Int. J. Clin. Exp. Hypn. 53, 148–169. 10.1080/00207140590927572 16025733

[B39] Kupers R. Faymonville M. Laureys S. (2005). The cognitive modulation of pain: hypnosis- and placebo-induced analgesia. Prog. Brain Res. 150, 251–269. 10.1016/S0079-6123(05)50019-0 16186029

[B40] Landry M. Appourchaux K. Raz A. (2014). Elucidating unconscious processing with instrumental hypnosis. Front. Psychol. 5:785. 10.3389/fpsyg.2014.00785 25120504 PMC4112913

[B41] Landry M. Lifschitz M. Raz A. (2017). Brain correlates of hypnosis: a systematic review and meta-analytic exploration. Neurosci. Biobehav. Rev. 81, 75–98. 10.1016/j.neubiorev.2017.02.020 28238944

[B42] Liang S. Choi K. Qin J. Wang Q. Heng P. (2016). Improving the discriination of hand motor imagery via virtual reality based visual guidance. Comput. Methods Programs Biomed. 132, 63–94. 10.1016/j.cmpb.2016.04.023 27282228

[B43] Lipari S. Baglio F. Griffanti L. Mendozzi L. Garegnani M. Motta A.. (2011). Altered and asymmetric default mode network activity in a “hypnotic virtuoso”: an fMRI and EEG study. Conscious. Cogn. 21, 393–400. 10.1016/j.concog.2011.11.006 22178091

[B44] Liu K. Chan C. Lee T. Hui-Chan C. (2004). Mental imagery for promoting relearning for people after stroke: a randomized controlled trial. Arch. Phys. Med. Rehabil. 85, 1403–1408. 10.1016/j.apmr.2003.12.035 15375808

[B45] Loison B. Moussaddaq A. Cormier J. Richard I. Ferrapie A. Ramond A.. (2013). Translation and validation of the french movement imagery questionnaire–revised second version (MIQ-RS). Ann. Phys. Rehabil. Med. 56, 157–173. 10.1016/j.rehab.2013.01.001 23415992

[B46] London P. Hart J. Leibovitz M. (1968). Eeg alpha rhythms and susceptibility to hypnosis. Nature 219, 71–2. 5659621 10.1038/219071a0

[B47] Malrewicz J. A. Godin J. Milton H. (1986). Erickson: de l'Hypnose Clinique à la Psychothérapie Stratégique. Paris.

[B48] Manly B. (2006). The generation of random permutations, in Randomization, Bootstrap and Monte Carlo Methods in Biology (Boca Raton, FL: Chapman & Hall/CRC).

[B49] Mcgeown W. Mazzoni G. Venneri A. Kirsch I. (2009). Hypnotic induction decreases anterior default mode activity. Conscious. Cogn. 18, 848–855. 10.1016/j.concog.2009.09.001 19782614

[B50] Moretti D. Babiloni F. Carducci F. Cincotti F. Remondini E. Rossini P.. (2003). Computerized processing of eeg–eog–emg artifacts for multi-centric studies in EEG oscillations and event-related potentials. Int. J. Psychophysiol. 47, 199–216. 10.1016/s0167-8760(02)00153-8 12663065

[B51] Mozenter R. H. Kurtz R. M. (1992). Prospective time estimation and hypnotizability in a simulator design. Int. J. Clin. Exp. Hypn. 40, 169–179. 10.1080/00207149208409655 1399154

[B52] Müller G. R. Neuper C. Rupp R. Keinrath C. Gerner H. J. Pfurtscheller G. (2003). Event-related beta EEG changes during wrist movements induced by functional electrical stimulation of forearm muscles in man. Neurosci. Lett. 340, 143–7. 10.1016/s0304-3940(03)00019-3 12668257

[B53] Muller K. Bacht K. Prochnow D. Schramm S. J.Seitz R. (2013). Activation of thalamus in motor imagery results from gating by hypnosis. Neuroimage 66, 361–367. 10.1016/j.neuroimage.2012.10.073 23128080

[B54] Muller K. Bacht K. Schramm S. Seitz R. (2012). The facilitating effect of clinical hypnosis on motor imagery: an fmri study. Behav. Brain Res. 231, 164–169. 10.1016/j.bbr.2012.03.013 22465168

[B55] Naish P. (2003). The production of hypnotic time-distortion: Determining the necessary conditions. Contemp. Hypn. 20, 3–15. 10.1002/ch.260

[B56] Nakayashiki K. Saeki M. Takata Y. Hayashi Y. Kondo T. (2014). Modulation of event-related desynchronization during kinematic and kinetic hand movements. J. Neuroeng. Rehab. 11:90. 10.1186/1743-0003-11-90 24886610 PMC4077682

[B57] Neuper C. Scherer R. Reiner M. Pfurtscheller G. (2005). Imagery of motor actions: differential effects of kinesthetic and visual–motor mode of imagery in single-trial {EEG}. Cogn. Brain Res. 25, 668–677. 10.1016/j.cogbrainres.2005.08.014 16236487

[B58] Neuper C. Wörtz M. Pfurtscheller G. (2006). ERD/ERS patterns reflecting sensorimotor activation and deactivation, in Event-Related Dynamics of Brain Oscillations, Vol. 159 of Progress in Brain Research, 1st edn, eds Neuper C. Klimesch W. (Amsterdam: Elsevier), 211–222. 10.1016/S0079-6123(06)59014-4 17071233

[B59] Oakley D. Halligan P. (2013). Hypnotic suggestion: opportunities for cognitive neuroscience. Nat. Rev. Neurosci. 14, 565–576. 10.1038/nrn3538 23860312

[B60] Perrin F. Pernier J. Betrand O. Echallier J. (1989). Spherical splines for scalp potential and current density mapping. Electroencephalogr. Clin. Neurophysiol. 72, 184–187. 2464490 10.1016/0013-4694(89)90180-6

[B61] Pfurtscheller G. (2001). Functional brain imaging based on ERD/ERS. Vis. Res. 41, 1257–1260. 10.1016/S0042-6989(00)00235-2 11322970

[B62] Pfurtscheller G. (2003). Induced oscillations in the alpha band: functional meaning. Epilepsia 44, 2–8. 10.1111/j.0013-9580.2003.12001.x 14641556

[B63] Pfurtscheller G. Aranibar A. (1977). Event-related cortical desynchronization detected by power measurment of the scalp EEG. Clin. Neurophysiol. 42, 817–826. 10.1016/0013-4694(77)90235-8 67933

[B64] Pfurtscheller G. Aranibar A. (1979). Evaluation of event-related desynchronization (ERD) preceding and following voluntary self-paced movement. Electroencephalogr. Clin. Neurophysiol. 46, 138–146. 86421 10.1016/0013-4694(79)90063-4

[B65] Pfurtscheller G. Lopes da Silva F. H. (1999). Event-related EEG/MEG synchronization and desynchronization: basic principles. Clin. Neurophysiol. 110, 1842–1857. 10576479 10.1016/s1388-2457(99)00141-8

[B66] Pfurtscheller G. Neuper C. (1997). Motor imagery activates primary sensorimotor area in humans. Neurosci. Lett. 239, 65–68. 9469657 10.1016/s0304-3940(97)00889-6

[B67] Pfurtscheller G. Neuper C. (2001). Motor imagery and direct brain-computer communication. Proc. IEEE 89, 1123–1134. 10.1109/5.939829

[B68] Pfurtscheller G. Neuper C. Brunner C. Lopes da Silva F. (2005). Beta rebound after different types of motor imagery in man. Neurosci. Lett. 378, 156–159. 10.1016/j.neulet.2004.12.034 15781150

[B69] Pfurtscheller G. Stancák A Jr Neuper C. (1996). Post-movement beta synchronization. A correlate of an idling motor area? Electroencephalogr. Clin. Neurophysiol. 98, 281–293. 8641150 10.1016/0013-4694(95)00258-8

[B70] Pfurtscheller G. Stancák A Jr. Edlinger G. (1997). On the existence of different types of central beta rhythms below 30 hz. Electroencephalogr. Clin. Neurophysiol. 102, 316–325. 9146493 10.1016/s0013-4694(96)96612-2

[B71] Pichiorri F. Morone G. Petti M. Toppi J. Pisotta I. Molinari M.. (2015). Brain–computer interface boosts motor imagery practice during stroke recovery. Ann. Neurol. 77, 851–865. 10.1002/ana.24390 25712802

[B72] Quiroga R. Garcia H. (2003). Single-trial event-related potentials with wavelet denoising. Clin. Neurophysiol. 114, 376–390. 10.1016/S1388-2457(02)00365-6 12559247

[B73] Raginsky B. (1963). Creativity in hypnosis. Psychosomatics 4, 170–172. 13990452 10.1016/s0033-3182(63)72548-5

[B74] Rainville P. Hofbauer R. Bushnell M. Duncan G. Price D. (2002). Hypnosis modulates activity in brain sutructures involved in the regulation of consciousness. J. Cogn. Neurosci. 14, 887–901. 10.1162/089892902760191117 12191456

[B75] Raz A. Shapiro T. Fan J. Posner M. (2003). Hypnotic suggestion and the modulation of stroop interference. Arch. Gen. Psychiatry 59, 1155–1161. 10.1001/archpsyc.59.12.1155 12470132

[B76] Rimbert S. Al-Chwa R. Zaepffel M. Bougrain L. (2018). Electroencephalographic modulations during an open- or closed-eyes motor task. PeerJ 6:e4492. 10.7717/peerj.4492 29576963 PMC5857351

[B77] Rimbert S. Avilov O. Adam P. Bougrain L. (2019a). Can suggestive hypnosis be used to improve Brain-Computer Interface performance? in 8th Graz Brain-Computer Interface Conference 2019 (Graz).

[B78] Rimbert S. Riff P. Gayraud N. Schmartz D. Bougrain L. (2019b). Median nerve stimulation based BCI: a new approach to detect intraoperative awareness during general anesthesia. Front. Neurosci. 13:622. 10.3389/fnins.2019.00622 31275105 PMC6593137

[B79] Roelofs K. Hoogduin K. Keijsers G. (2002). Motor imagery during hypnotic arm paralysis in high and low hypnotizable subjects. Int. J. Clin. Exp. Hypn. 50, 51–66. 10.1080/00207140208410090 11783441

[B80] Salem G. Bonvin E. (2012). Soigner par l'Hypnose, 6th Edn. Paris: Masson.

[B81] Salenius S. Schnitzler A. Salmelin R. Jousmäki V. Hari R. (1997). Modulation of human cortical rolandic rhythms during natural sensorimotor tasks. Neuroimage 5, 221–228. 9345551 10.1006/nimg.1997.0261

[B82] Sharma N. Pomeroy V. Baron J. (2006). Motor imagery: a backdoor to the motor system after stroke? Stroke 37, 1941–1952. 10.1161/01.STR.0000226902.43357.fc 16741183

[B83] Simpkins C. Simpkins M. A. (2012). Mindfulness and hypnosis: the power of suggestion to transform experience by M. Yapko. Am. J. Clin. Hypn. 55, 199–200. 10.1080/00029157.2013.686417

[B84] Spiegel H. Spiegel D. (1978). Trance and Treatment: Clinical Uses of Hypnosis. New York, NY: Basic Books, Inc.

[B85] Takarada Y. Nozaki D. (2014). Hypnotic suggestion alters the state of the motor cortex. Neurosci. Res. 85, 28–32. 10.1016/j.neures.2014.05.009 24973620

[B86] Tan L.-F. Dienes Z. Jansari A. Goh S.-Y. (2014). Effect of mindfulness meditation on brain–computer interface performance. Conscious. Cogn. 23, 12–21. 10.1016/j.concog.2013.10.010 24275085

[B87] Verleger R. (1993). Valid identification of blink artefacts: are they larger than 50 v in EEG records? Electroencephalogr. Clin. Neurophysiol. 87:354–363. 10.1016/0013-4694(93)90148-O 7508367

[B88] Wood C. Bioy A. (2008). Hypnosis and pain in children. J. Pain Symptom Manage. 35, 437–446. 10.1016/j.jpainsymman.2007.05.009 18243640

[B89] World Medical A. (2002). World Medical Association Declaration of Helsinki: ethical principles for medical research involving human subjects. J. Postgrad. Med. 48, 206–208. 10.1001/jama.2013.281053 KIE: KIE Bib: human experimentation. 12432198

[B90] Wright M. (1960). Hypnosis and rehabilitation. Rehabil. Lit. 21, 2–12. 13846094

[B91] Yuksel R. Ozcan O. Dane S. (2013). The effects of hypnosis on heart rate variability. Int. J. Clin. Exp. Hypn. 61, 162–171. 10.1080/00207144.2013.753826 23427840

[B92] Zimmermann-Schlatter A. Schuster C. Puhan M. Siekierka E. Steurer J. (2008). Efficiacy of motor imagery in post-stroke rehabilitation: a systematic review. J. Neuroeng. Rehabil. 14, 5–8. 10.1186/1743-0003-5-8 PMC2279137 18341687

