# Supplementary figures and images for: Hypnotic State Modulates Sensorimotor Beta Rhythms During Real Movement and Motor Imagery
Source: Front Psychol. 2019 Oct 22;10:2341. doi: 10.3389/fpsyg.2019.02341 (PMC6817584; doi:10.3389/fpsyg.2019.02341)

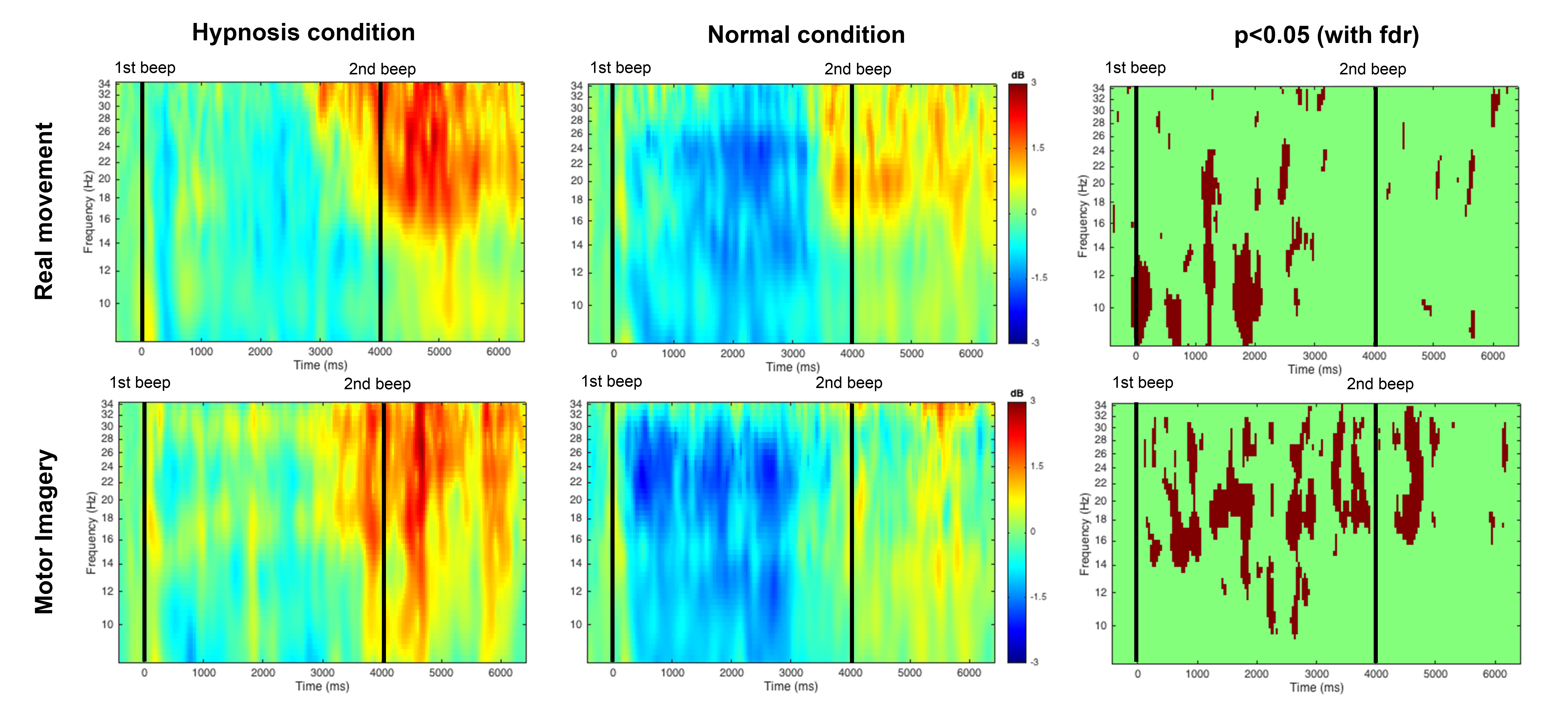

Supplement: Figure S1 — Time-frequency grand average with Laplacian filter analysis corresponding to an event-related spectral perturbation (ERSP) for Session 1 (Normal condition) and Session 2 (Hypnotic condition) for a real movement and a motor imagery for electrode C3. Red corresponds to a strong ERS and blue to a strong ERD. Significant differences (p < 0.05) with a False Discovery Rate (FDR) correction are shown in the right part of the figure. [file Image_1.JPEG]

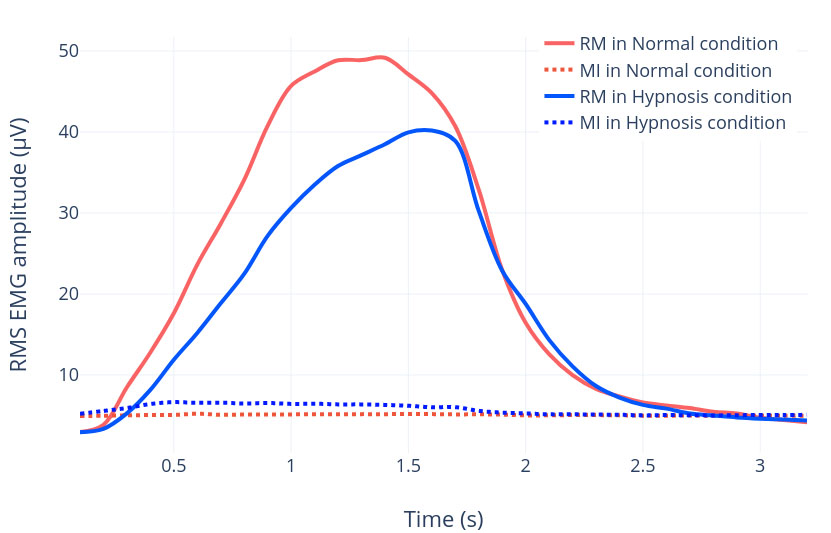

Supplement: Figure S2 — The root-mean-square (RMS) envelope of the EMG signal for real movement and motor imagery (dashed line) during both hypnosis (in blue) and the normal condition (in red). [file Image_2.JPEG]

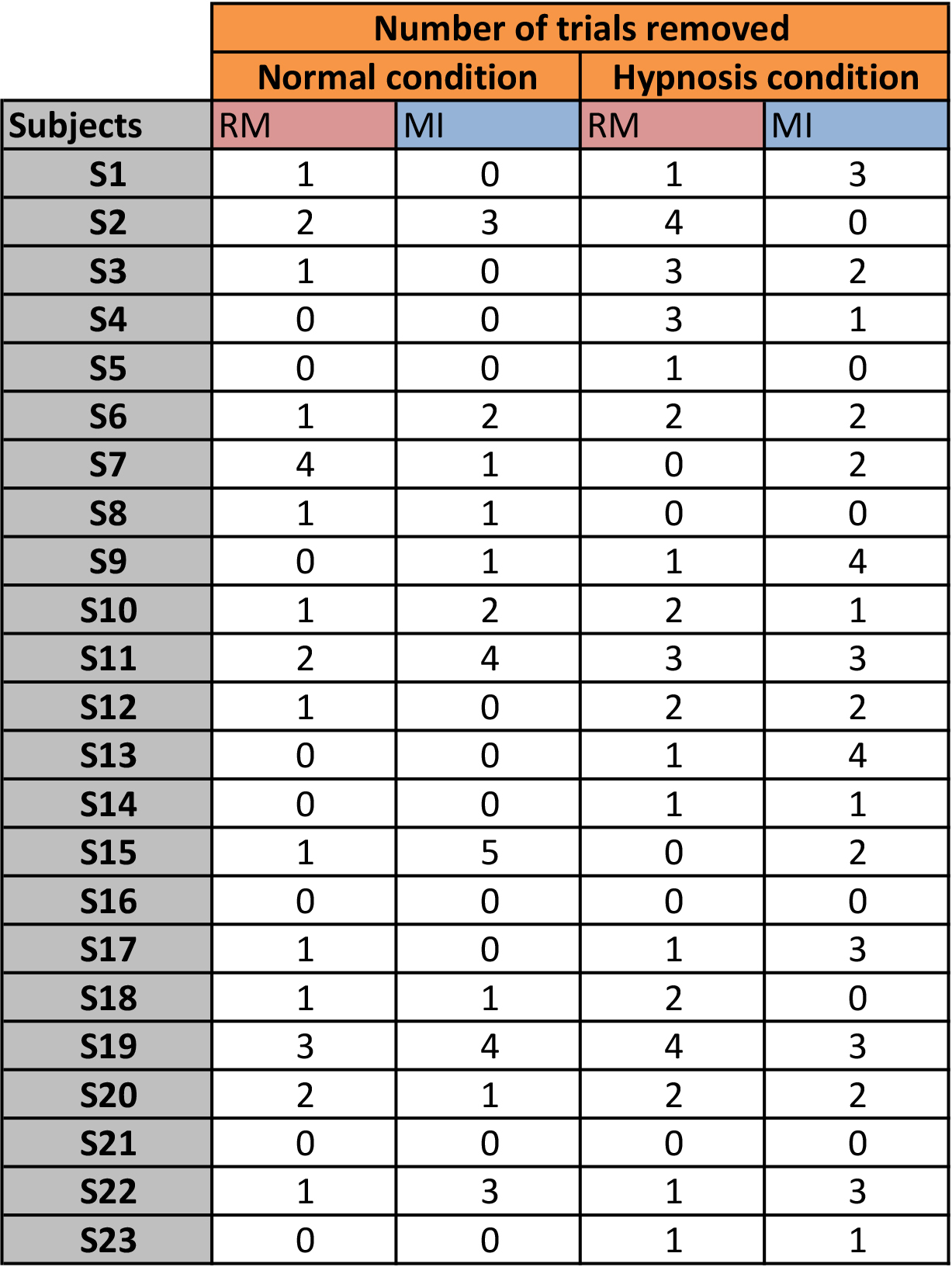

Supplement: Figure S3 — Number of trials removed for each subject for real movement and motor imagery during hypnosis and normal condition. [file Image_3.JPEG]
